# Altered Expression of Epigenetic and Transcriptional Regulators in ERβ Knockout Rat Ovaries During Postnatal Development

**DOI:** 10.3390/ijms26020760

**Published:** 2025-01-17

**Authors:** Kevin Vo, Yashica Sharma, V. Praveen Chakravarthi, Ryan Mohamadi, Elizabeth S. Bahadursingh, Amelia Mohamadi, Vinesh Dahiya, Cinthia Y. Rosales, Grace J. Pei, Patrick E. Fields, M. A. Karim Rumi

**Affiliations:** Department of Pathology and Laboratory Medicine, University of Kansas Medical Center, Kansas City, KS 66160, USA; kvo5@kumc.edu (K.V.); yashica2025@gmail.com (Y.S.); praghavulu@kumc.edu (V.P.C.); ryanm700@gmail.com (R.M.); elizabeth.bahadursingh@ucdconnect.ie (E.S.B.); amelia.mohamadi@ku.edu (A.M.); vinesh.dahiyampharm@gmail.com (V.D.); cinthia_0630@yahoo.com (C.Y.R.); pfields@kumc.edu (P.E.F.)

**Keywords:** ovarian follicles, postnatal development, transcriptome, epigenetic regulators, transcription factors, estrogen receptor β, deletion mutants

## Abstract

We analyzed the transcriptome data of wildtype and estrogen receptor β knockout (*Erβ^KO^*) rat ovaries during the early postnatal period and detected remarkable changes in epigenetic regulators and transcription factors. Compared with postnatal day (PD) 4.5 ovaries, PD 6.5 wildtype ovaries possessed 581 differentially expressed downstream transcripts (DEDTs), including 17 differentially expressed epigenetic regulators (DEERs) and 23 differentially expressed transcription factors (DETFs). Subsequently, compared with PD 6.5 ovaries, PD 8.5 wildtype ovaries showed 920 DEDTs, including 24 DEERs and 68 DETFs. The DEDTs, DEERs, and DETFs in wildtype ovaries represented the gene expression during primordial follicle activation and the gradual development of primary follicles of first-wave origin because the second-wave follicles remained dormant during this developmental period. When we compared the transcriptome data of age-matched *Erβ^KO^* ovaries, we observed that PD 6.5 *Erβ^KO^* ovaries had 744 DEDTs compared with PD 4.5 ovaries, including 46 DEERs and 55 DETFs. The loss of ERβ rapidly activated the primordial follicles of both first- and second-wave origin on PD 6.5 and showed a remarkable increase in DEDTs (744 vs. 581). However, compared with PD 6.5 ovaries, PD 8.5 *Erβ^KO^* ovaries showed only 191 DEDTs, including 8 DEERs and 10 DETFs. This finding suggests that the PD 8.5 *Erβ^KO^* ovaries did not undergo remarkable ovarian follicle activation greater than that had already occurred in PD 6.5 *Erβ^KO^* ovaries. The results also showed that the numbers of DEERs and DETFs were associated with increased changes in DEDTs; the greater the number of DEERs or DETFs, the larger the number of DEDTs. In addition to the quantitative differences in DEERs and DETFs between the wildtype and *Erβ^KO^* ovaries, the differentially expressed regulators showed distinct patterns. We identified that 17 transcripts were tied to follicle assembly, 6 to follicle activation, and 12 to steroidogenesis. Our observations indicate that a loss of ERβ dysregulates the epigenetic regulators and transcription factors in *Erβ^KO^* ovaries, which disrupts the downstream genes in ovarian follicles and increases follicle activation. Further studies are required to clarify if ERβ directly or indirectly regulates DEDTs, including DEERs and DETFs, during the neonatal development of rat ovarian follicles.

## 1. Introduction

Ovarian follicles are the functional units of the ovary that accomplish steroidogenesis and oogenesis [1]. The first step in ovarian follicle development is oocyte nest breakdown coupled with primordial follicle assembly [2,3]. Primordial follicles in the mouse or rat ovary are formed into two distinct waves [3,4,5,6]. The first wave of primordial follicles develops in the ovarian medulla at birth and is rapidly activated [3,4,5,6]. In contrast, the second wave forms during postnatal days (PDs) 4.5 to 8.5 and remains dormant [3,4,5,6,7]. The dormant follicles of the second wave establish the ovarian reserve for the entire reproductive period and determine ovarian longevity [3,5]. In contrast, first-wave follicles are activated immediately after assembly, and their roles remain largely unclear [4,5]. It has been shown that two distinct pathways of pregranulosa cells support the two different waves of ovarian follicles [8]. The granulosa cells of the FWFs express genes different from those of the SWFs [7].

Estrogen receptor β (ERβ) is the predominant transcription factor in granulosa cells, and the expression of ERβ is very high in the ovary compared with other tissues [9]. Studies have demonstrated that the loss of ERβ results in ovulation failure in mice and rats [10,11,12]. Previous studies have identified that ERβ regulates an important group of genes essential for preovulatory follicle maturation [11,12,13]. ERβ is essential for preovulatory follicle maturation from the antral stage, and the ovaries of *Erβ^KO^* mice or rats contain fewer large antral follicles [11,13,14]. The loss of ERβ reduces the expression of *Cyp19a1*, *Lhcgr*, and *Ptgs2* and increases the expression of Ar in antral follicles [14,15]. In addition to the steroidogenic genes, a loss of ERβ signaling also affects oocyte maturation [16]. Although numerous studies have reported the role of ERβ in preovulatory follicle maturation [11,13,14], several studies have shown its role in oocyte nest breakdown [17,18,19]. The activation of ERβ in neonatal mice with diethylstilbestrol (DES) and other agonists resulted in polyovular follicles in mice [20,21,22]. We observed increased primordial follicle activation due to the loss of ERβ in mutant rats, which was evident as early as PD 4.5 [7,23]. A loss-of-function mutation in the ERβ gene in humans was also found to cause complete ovarian failure [24]. However, a few studies have analyzed follicular genes that were altered due to the loss of ERβ, particularly during early ovarian development [7,15,21].

This study utilized wildtype and *Erβ^KO^* ovaries collected on PD 4.5, 6.5, and 8.5, the crucial timepoints of oocyte nest breakdown, primordial follicle formation, and primordial follicle activation. Total RNA was purified, and transcriptome analyses were performed using RNA sequencing. We observed remarkable differential expressions in epigenetic regulators, transcription factors, and downstream transcripts. We analyzed each gene’s differentially expressed transcript variants to detect the actual mRNAs that underwent up- or downregulation [25]. The results indicate that a loss of ERβ dysregulates the epigenetic regulators and transcription factors in *Erβ^KO^* ovaries, disrupting the downstream transcripts and increasing ovarian follicle activation.

## 2. Results

In this study, we performed transcript variant analyses instead of gene expression analyses that imply one gene expresses one mRNA [25]. This approach was taken to achieve greater accuracy in the gene expression analyses [25]. The transcript variants were named according to the Ensembl reference sequence nomenclature (mRatBN7.2.111). We initially compared the RNA-Seq data of PD 4.5, 6.5, and 8.5 *Erβ^KO^* ovaries with those of age-matched wildtype ovaries and identified the differentially expressed epigenetic regulators (DEERs), differentially expressed transcription factors (DETFs), and differentially expressed downstream transcripts (DEDTs) due to the loss of ERβ. In the next step, we identified the changes in epigenetic regulators, transcription factors, and downstream transcripts during postnatal development in wildtype and *Erβ^KO^* rat ovaries. According to recent publications, the expression of 690 epigenetic regulators and 1374 transcription factors was investigated [26,27]. This was performed by comparing the RNA-Seq data of PD 6.5 with those of PD 4.5, and by comparing the RNA-Seq data of PD 8.5 with those of PD 6.5 for the same genotype. We also compared the identities of the DEERs, DEDTs, and DEDTs to ascertain if their functional involvement in steroidogenesis or folliculogenesis was already known.

### 2.1. Changes in Epigenetic Regulators Due to the Loss of ERβ

**Figure 1:**
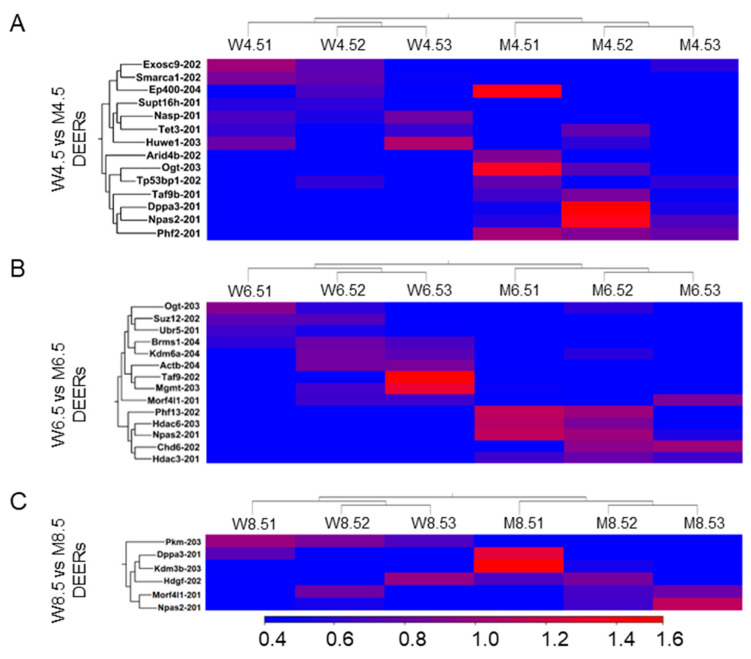
Heat maps showing differentially expressed epigenetic regulators (DEERs) due to the loss of ERβ. RNA-Seq analysis was performed for postnatal days (PDs) 4.5, 6.5, and 8.5 wildtype (W) and *Erβ^KO^* (M) rat ovaries (**A**–**C**). The three left columns represent wildtype data, and the three right columns represent *Erβ^KO^* data. The heat maps show DEERs in PD 4.5 (**A**), PD 6.5 (**B**), and PD 8.5 (**C**) *Erβ^KO^* ovaries compared with age-matched wildtype ovaries (≥2 absolute fold changes; FDR *p* ≤ 0.05; TPM ≥ 5). All DEERs represent the TPM values of the transcript variants.

Compared with PD 4.5 wildtype ovaries, PD 4.5 *Erβ^KO^* ovaries showed the differential expression of 14 epigenetic regulators (7 upregulated and 7 downregulated) (≥2 absolute fold changes and FDR of *p* ≤ 0.05; TPM values ≥ 5) (Figure 1A). Compared with PD 6.5 wildtype ovaries, PD 6.5 *Erβ^KO^* ovaries also showed 14 DEERs (5 upregulated and 9 downregulated) (Figure 1B). However, compared with PD 8.5 wildtype ovaries, PD 8.5 *Erβ^KO^* ovaries had only 6 DEERs (1 downregulated and 5 upregulated). Remarkably, histone reader *Morf4l1-201* was significantly downregulated in PD 6.5 *Erβ^KO^* ovaries but upregulated in PD 8.5 *Erβ^KO^* ovaries (Figure 1C). The upregulated DEERs in PD 4.5, 6.5, and 8.5 *Erβ^KO^* ovaries included *Npas2-201*, *Dppa3-201*, and *Taf9b-201*, whereas the downregulated DEERs in PD 4.5, 6.5, and 8.5 included *Exocsc9-202*, *Brms1-204*, *Actb-204*, and *Pkm-203*.

### 2.2. Changes in Transcription Factors Due to the Loss of ERβ

**Figure 2:**
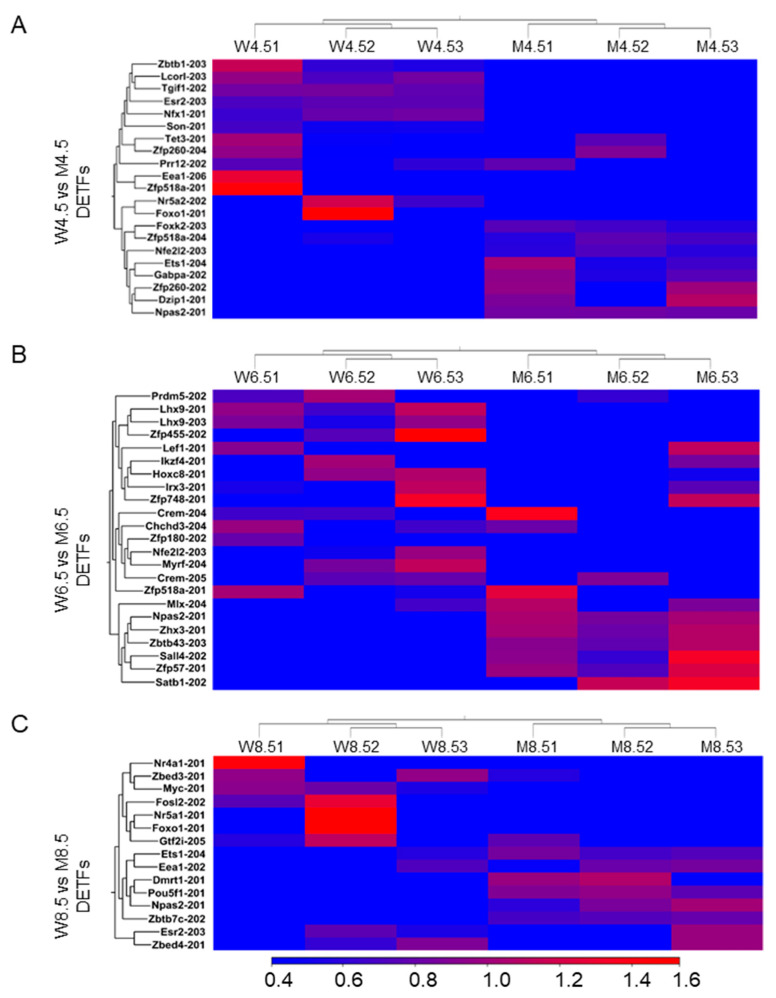
Heat maps showing differentially expressed transcription factors (DETFs) due to the loss of ERβ. RNA-Seq analysis was performed for postnatal days (PDs) 4.5, 6.5, and 8.5 wildtype (W) and *Erβ^KO^* (M) ovaries (**A**–**C**). The three left columns represent wildtype data, and the three right columns represent *Erβ^KO^* data. The heat maps show DETFs in PD 4.5 (**A**), PD 6.5 (**B**), and PD 8.5 (**C**) *Erβ^KO^* ovaries compared with age-matched wildtype ovaries (≥2 absolute fold changes; FDR *p* ≤ 0.05; TPM ≥ 5). All DETFs represent the TPM values of the transcript variants.

Unlike DEERs, the majority of DETFs (≥2 absolute fold changes and an FDR of *p* ≤ 0.05; TPM values ≥ 5) were downregulated in *Erβ^KO^* ovaries at all timepoints. Compared with age-matched wildtype ovaries, PD 4.5 *Erβ^KO^* ovaries had 21 DETFs (13 downregulated) (Figure 2A), PD 6.5 *Erβ^KO^* ovaries had 23 DETFs (16 downregulated) (Figure 2B), and PD 8.5 *Erβ^KO^* ovaries had 15 DETFs (9 downregulated) (Figure 2C). Notably, the upregulated DETFs were *Zfp260-202* in PD 4.5 ovaries, *Mlx-204* in PD 6.5 ovaries, and *Eea1-202* in PD 8.5 *Erβ^KO^* ovaries. The downregulated DETFs were *Prr12-202* in PD 4.5 ovaries, *Lhx9-201* in PD 6.5 ovaries, and *Zbed3-201* in PD 8.5 *Erβ^KO^* ovaries.

### 2.3. Changes in Downstream Transcripts Due to the Loss of ERβ

**Figure 3:**
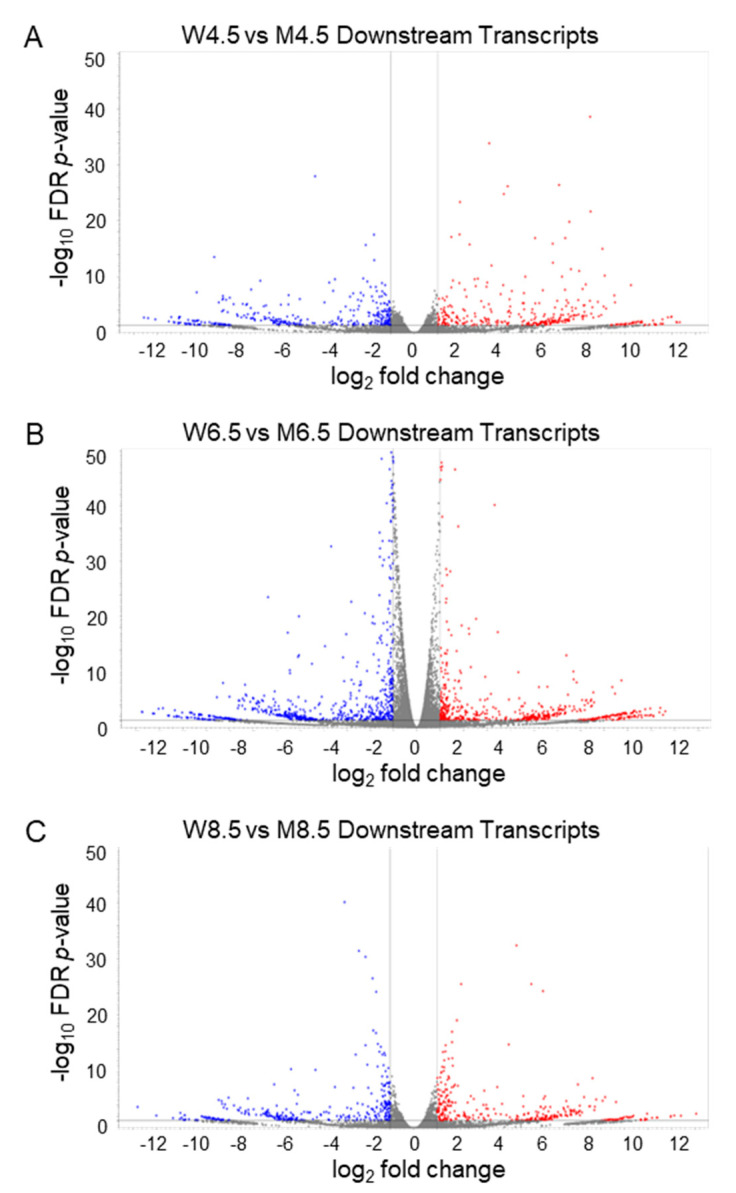
Volcano plots of differentially expressed downstream transcripts for wildtype (W) and *Erβ^KO^* (M) ovaries. Volcano plots displaying the differentially expressed downstream transcripts (DEDTs) of wildtype and *Erβ^KO^* ovaries on postnatal days (PDs) 4.5 (**A**), 6.5 (**B**), and 8.5 (**C**). All DEDTs (≥2 absolute fold changes; FDR *p* ≤ 0.05; TPM values ≥ 5) represent the relative TPM values of the transcript variants. The red dots denote upregulation, and the blue dots denote downregulation of DEDTs. FDR: false discovery rate.

Compared with PD 4.5 wildtype ovaries, age-matched PD *Erβ^KO^* ovaries had 346 DEDTs (178 upregulated) (Figure 3A). However, PD 6.5 ovaries had 581 DEDTs (203 upregulated) (Figure 3B) and PD 8.5 ovaries had 348 DEDTs (180 upregulated) (Figure 3C). Although a transcript like *Pam-204* was significantly repressed in *Erβ^KO^* ovaries at all timepoints, *Morf4l1-201* underwent dramatic changes; it was not expressed at PD 4.5, was repressed at PD 6.5, and was highly upregulated at PD 8.5 in *Erβ^KO^* ovaries. The remarkably changed DEDTs included *Smc3-201* and *Lrrc8d-205*, downregulated at PD 4.5 but upregulated at PD 6.5, and remained unchanged in PD 8.5 *Erβ^KO^* ovaries.

### 2.4. Changes in Epigenetic Regulators During Postnatal Development

**Figure 4:**
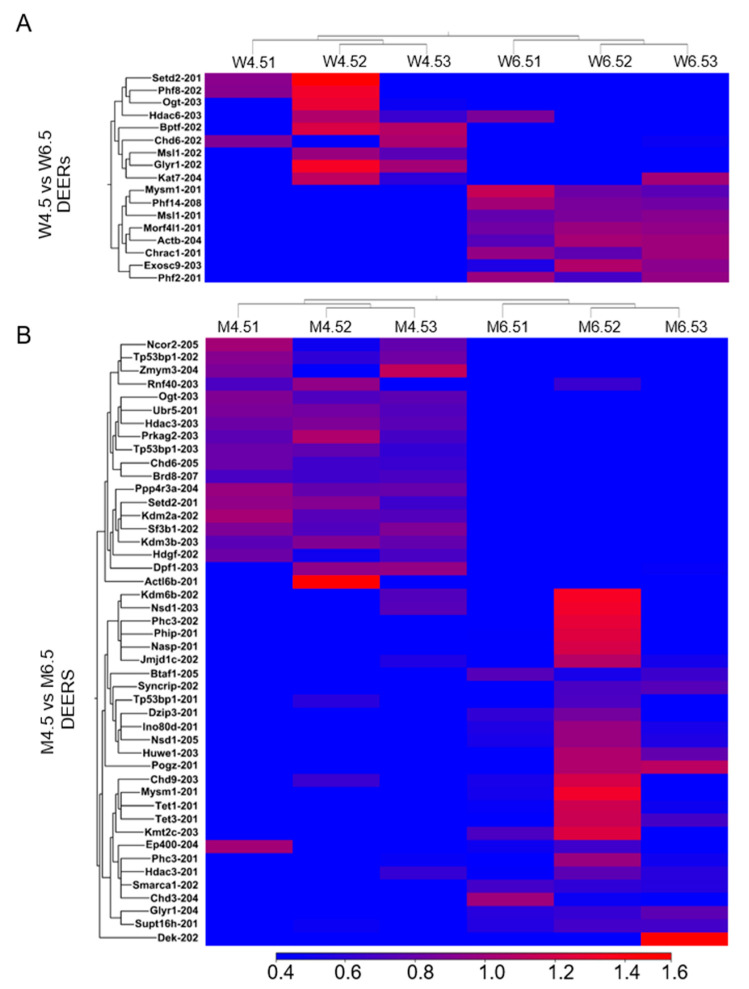
Heat maps showing differentially expressed epigenetic regulators (DEERs) in postnatal day (PD) 6.5 wildtype (W) and *Erβ^KO^* (M) rat ovaries. RNA-Seq analysis was performed for PD 4.5 and PD 6.5 wildtype (W) and *Erβ^KO^* (M) rat ovaries. The three left columns represent PD 4.5 data, and the three right columns represent PD 6.5 data. (**A**) Heat maps showing DEERs (≥2 absolute fold changes; TPM values ≥ 5; FDR *p* ≤ 0.05) of PD 6.5 wildtype ovaries compared with PD 4.5 ovaries. (**B**) Heat maps showing DEERs of PD 6.5 *Erβ^KO^* ovaries compared with PD 4.5 *Erβ^KO^* ovaries. All DEERs represent the relative TPM values of the transcript variants.

All epigenetic regulators, transcription factors, and downstream transcripts were analyzed based on the TPM (transcript per million) values of the transcript variants [25]. Compared with PD 4.5 wildtype ovaries, PD 6.5 wildtype ovaries showed the upregulation of 8 out of 17 differentially expressed epigenetic regulators (DEERs) (≥2 absolute fold changes and an FDR of *p* ≤ 0.05; TPM values ≥ 5) (Figure 4A). The upregulated DEERs included histone readers *Phf14-208* and *Morf4l1-201*, histone writer *Msl1-201*, histone chaperone *Chrac1-201*, and histone erasers *Phf2-201* and *Mysm1-201* (Supplementary Table S1-1). In contrast, the downregulated DEERs included histone reader *Glyr1-202*; histone writers *Setd2-201*, *Msl1-202*, *Kat7-204*, and *Ogt-203*; and histone erasers *Phf8-202* and *Hdac6-203* (Supplementary Table S1-1). Although chromatin remodeler *Actb-204* was upregulated, *Bptf-202* and *Chd6-202* were downregulated in PD 6.5 wildtype ovaries (Supplementary Table S1-1).

Compared with PD 4.5 *Erβ^KO^* ovaries, PD 6.5 *Erβ^KO^* ovaries showed upregulation of 27 out of 46 DEERs (Figure 4B). The upregulated DEERs included histone readers *Supt16h-201*, *Pogz-201*, and *Glyr1-204*; histone writers *Kmt2c-203*, *Huwe1-203*, *Dzip3-201*, and *Nsd1-205*; and histone erasers *Hdac3-201*, *Jmjd1c-202*, and *Mysm1-201* (Supplementary Table S1-2). In contrast, the downregulated DEERs included histone readers *Tp53bp1-202*, *Tp53bp1-203*, and *Brd8-207*; histone writers *Setd2-201*, *Rnf40-203*, *Prkag2-203*, and *Ogt-203*; and histone erasers *Kdm2a-202*, *Kdm3b-203*, *Ncor2-205*, *Ppp4r3a-204*, *Hdac3-203*, and *Zmym3-204* (Supplementary Table S1-2). Chromatin remodelers *Smarca1-202*, *Btaf1-205*, and *Ino80d-201* were upregulated in PD 6.5 ovaries and *Dpf1-203* and *Ubr5-201* were downregulated (Supplementary Table S1-2).

**Figure 5:**
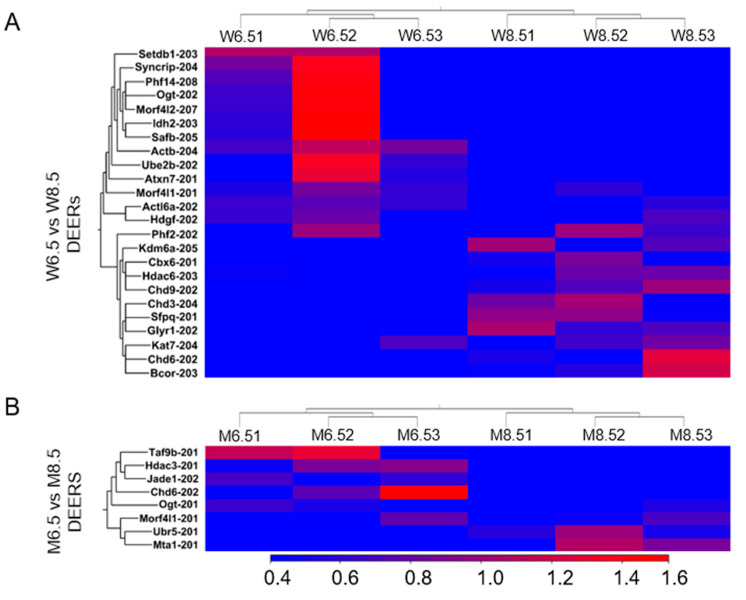
Heat maps showing differentially expressed epigenetic regulators (DEERs) in postnatal day (PD) 8.5 wildtype (W) and *Erβ^KO^* (M) ovaries. RNA-Seq analysis was performed for PD 6.5 and 8.5 wildtype and *Erβ^KO^* rat ovaries. The three left columns represent PD 6.5 data, and the three right columns represent PD 8.5 data. (**A**) Heat maps showing DEERs (≥2 absolute fold changes; FDR *p* ≤ 0.05; TPM values ≥ 5) of PD 8.5 wildtype ovaries compared with PD 6.5 wildtype ovaries. (**B**) Heat maps showing DEERs of PD 8.5 *Erβ^KO^* ovaries compared with PD 6.5 *Erβ^KO^* ovaries. All DEERs represent the relative TPM values of the transcript variants.

We also detected that PD 8.5 wildtype ovaries exhibited an upregulation of 11 out of 24 DEERs compared with PD 6.5 wildtype ovaries (Figure 5A). The upregulated DEERs included histone readers *Glyr1-202* and *Cbx6-201*, histone writer *Kat7-204*, and histone erasers *Hdac6-203* and *Kdm6a-205*. The downregulated DEERs included histone readers *Morf4l1-201* and *Phf14-208*; histone writers *Atxn7-201*, *Setdb1-203*, *Ube2b-202*, and *Ogt-202*; and histone eraser *Morf4l2-207* (Supplementary Table S1-3). We also identified that chromatin remodelers *Sfpq-201*, *Chd3-204*, and *Chd9-202* were upregulated in PD 8.5 wildtype ovaries, whereas *Actb-204*, *Actl6a-202*, and *Hdgf-202* were downregulated (Supplementary Table S1-3).

In contrast, compared with PD 6.5 *Erβ^KO^* ovaries, PD 8.5 *Erβ^KO^* ovaries showed the upregulation of 3 out of only 8 DEERs (Figure 5B), which included histone reader *Morf4l1-201* and chromatin remodelers *Ubr5-201* and *Mta1-201*. The downregulated DEERs included histone writer *Jade1-202*, histone chaperone *Taf9b-201*, histone eraser *Hdac3-20*, and chromatin remodeler *Chd6-202* (Supplementary Table S1-4).

### 2.5. Changes in Transcription Factors During Postnatal Development

**Figure 6:**
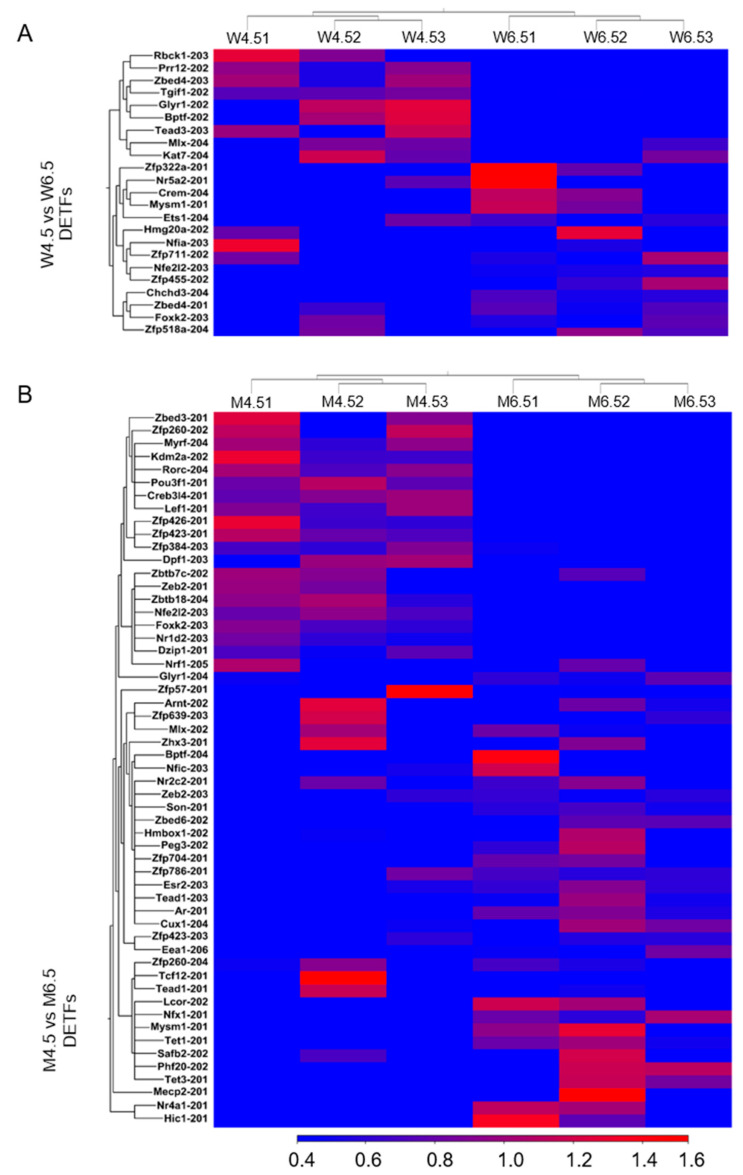
Heat maps showing differentially expressed transcription factors (DETFs) on postnatal day (PD) 6.5. RNA-Seq analysis was performed for PD 4.5 and PD 6.5 wildtype (W) and *Erβ^KO^* (M) rat ovaries. The three left columns represent PD 4.5 data, and the three right columns represent PD 6.5 data. (**A**) Heat maps showing DETFs (≥2 absolute fold changes; TPM values ≥ 5; FDR *p* ≤ 0.05) of PD 6.5 wildtype ovaries compared with those of PD 4.5. (**B**) Heat maps showing DETFs of PD 4.5 and PD 6.5 for the *Erβ^KO^* group ovaries. All DETFs represent the relative TPM values of the transcript variants.

We detected that 14 out of 23 differentially expressed transcription factors (DETFs) were upregulated in PD 6.5 wildtype ovaries compared with PD 4.5 ovaries (≥2 absolute fold changes and an FDR of *p* ≤ 0.05; TPM values ≥ 5) (Figure 6A). The top 5 upregulated DETFs included *Zfp518a-204*, *Foxk-203*, *Zfp711-202*, *Chchd3-204*, and *Zbed4-201* and the top 5 downregulated DETFs included *Mlx-204*, *Kat7-204*, *Tgif1-202*, *Prr12-202*, and *Rbck1-203* (Supplementary Table S2-1). Compared with PD 4.5 *Erβ^KO^* ovaries, PD 6.5 *Erβ^KO^* ovaries showed the upregulation of 36 out of 55 DETFs (Figure 6B). The top 5 upregulated DETFs included *Zfp786-201*, *Mlx-202*, *Nfx1-201*, *Glyr1-204*, and *Son-201* and the top 5 downregulated DETFs were *Zfp384-203*, *Zfp260-202*, *Zbtb7c-202*, *Dpf1-203*, and *Nr1d2-203* (Supplementary Table S2-2).

**Figure 7:**
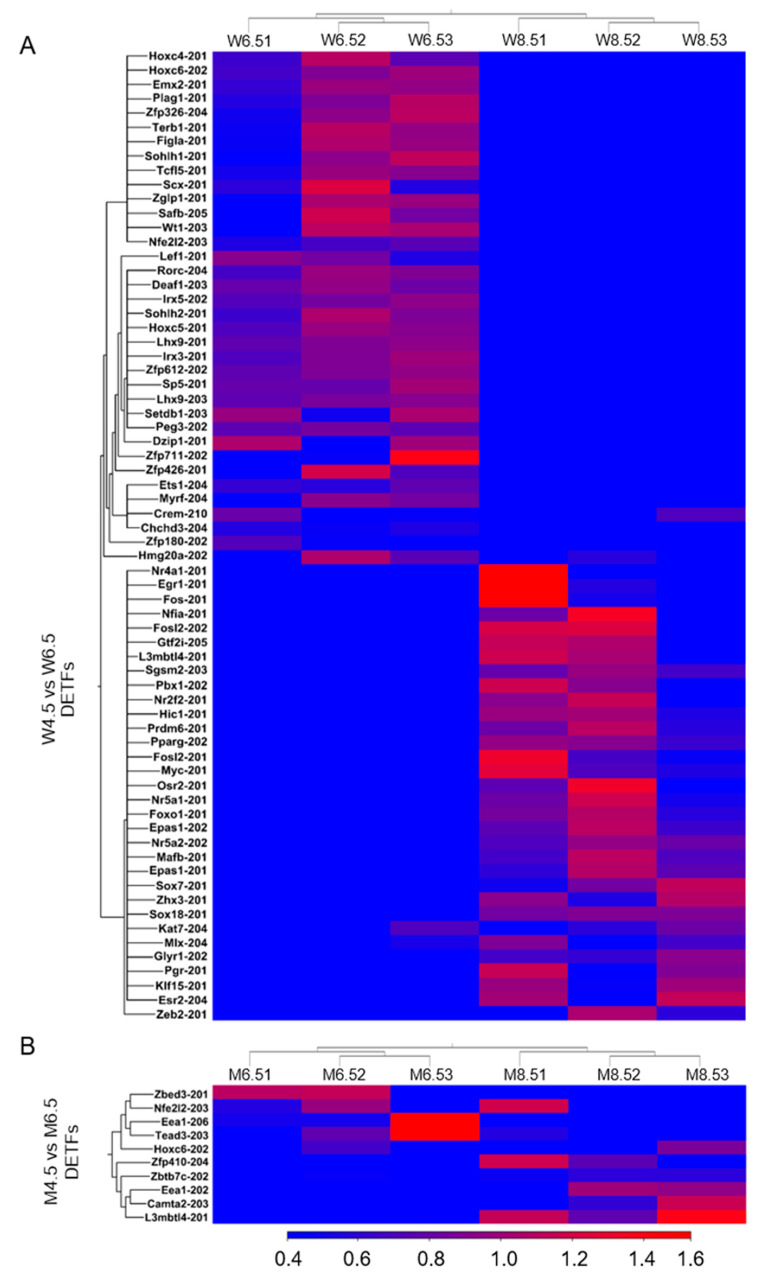
Heat maps showing differentially expressed transcription factors (DETFs) of postnatal day (PD) 8.5 ovaries. RNA-Seq analysis was performed for PD 6.5 and PD 8.5 wildtype (W) and *Erβ^KO^* (M) rat ovaries. The three left columns represent PD 6.5 data, and the three right columns represent PD 8.5 data. (**A**) Heat maps showing DETFs (≥2 absolute fold changes; TPM values ≥ 5; FDR *p* ≤ 0.05) of PD 8.5 wildtype ovaries compared with PD 6.5 ovaries. (**B**) Heat maps showing DETFs of PD 8.5 *Erβ^KO^* ovaries compared with PD 6.5 ovaries. All DETFs represent the relative TPM values of the transcript variants.

In contrast, 32 out of 68 DETFs were upregulated in PD 8.5 wildtype ovaries compared with PD 6.5 wildtype ovaries (Figure 7A). The top 5 upregulated DETFs were *Mlx-204*, *Kat7-204*, *Fosl2-201*, *Pparg-202*, and *Foxo1-201* and the top 5 downregulated DETFs were *Crem-210*, *Nfe2l2-203*, *Ets1-204*, *Lhx9-203*, and *Lhx9-201* (Supplementary Table S2-3). Compared with PD 6.5 *Erβ^KO^* ovaries, PD 8.5 *Erβ^KO^* ovaries showed 10 DETFs (Figure 7B). The upregulated DETFs were *Zbtb7c-202*, *Zfp410-204*, *Eea1-202*, *Camta2-203*, and *L3mbtl4-201* and the downregulated DETFs were *Nfe2l2-203*, *Zbed3-201*, *Tead3-203*, *Hoxc6-202*, and *Eea1-206* (Supplementary Table S2-4).

### 2.6. Changes in Downstream Transcripts During Postnatal Development

**Figure 8:**
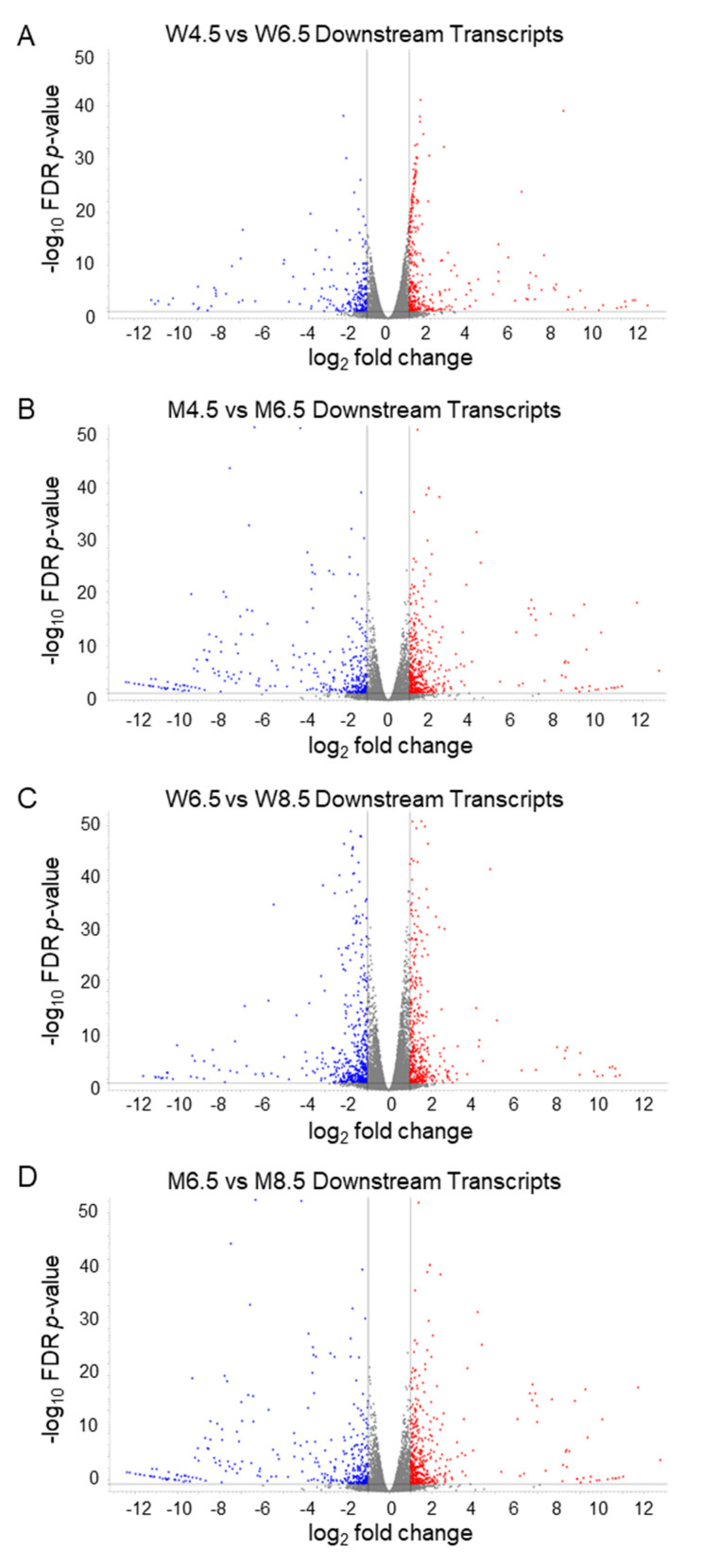
Volcano plots displaying differentially expressed downstream transcripts (DEDTs). Volcano plots showing DEDTs of postnatal day (PD) 6.5 wildtype (**A**) or PD 6.5 *Erβ^KO^* (**B**) ovaries compared with PD 4.5 wildtype (A) or PD 4.5 *Erβ^KO^* (B) ovaries. The other volcano plots show the DEDTs of PD 8.5 wildtype (**C**) or PD 8.5 *Erβ^KO^* (**D**) ovaries compared with PD 6.5 wildtype (**C**) or PD 6.5 *Erβ^KO^* (**D**) ovaries. All DEDTs (≥2 absolute fold changes; TPM values ≥ 5; FDR *p* ≤ 0.05) represent the relative TPM values of the transcript variants. The red dots denote upregulation, and the blue dots denote downregulation of DEDTs. W: wildtype; M: *Erβ^KO^*; FDR: false discovery rate.

Changes in the DEERs and DEFTs of PD 6.5 wildtype ovaries showed the upregulation of 368 out of 581 DEDTs compared with PD 4.5 ovaries (Figure 8A). Similarly, PD 6.5 *Erβ^KO^* ovaries upregulated 419 out of 744 DEDTs compared with PD 4.5 *Erβ^KO^* ovaries (Figure 8B). Although PD 8.5 wildtype ovaries upregulated 424 of 920 DEDTs compared with PD 6.5 ovaries (Figure 8C), PD 8.5 *Erβ^KO^* ovaries had significantly fewer DEDTs and 111 of 191 DEDTs were upregulated (Figure 8D). A selected group of differentially expressed DEDTs in wildtype and *Erβ^KO^* ovaries are shown in Supplementary Tables S3-1 to S3-4.

### 2.7. Comparisons of DEERs, DETFs, and DEDTs During Postnatal Development

**Figure 9:**
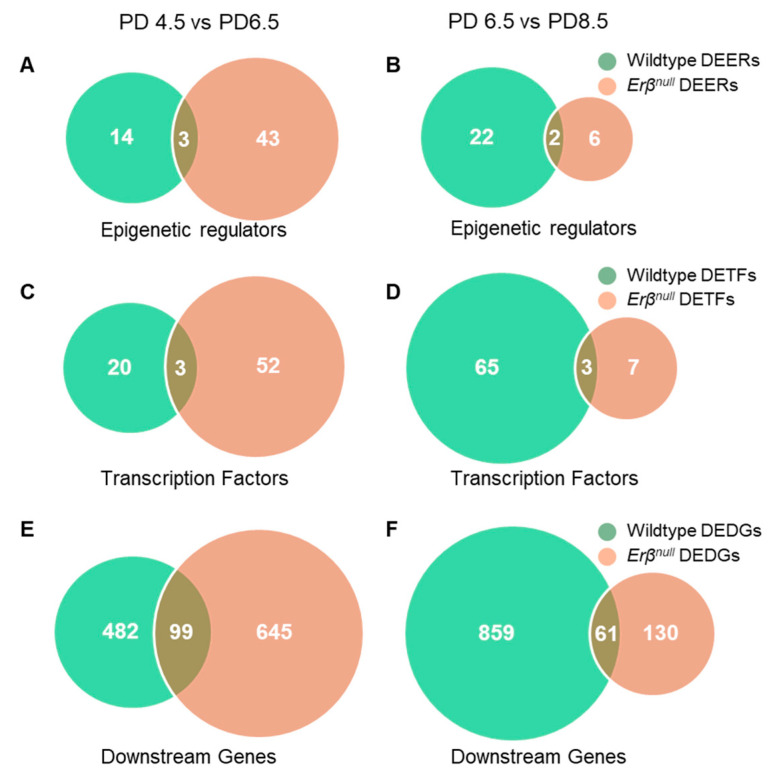
A comparison of differentially expressed epigenetic regulators (DEERs), transcription factors (DETFs), and downstream transcripts (DEDTs). Differential expression of DEERs, DETFs, and DEDTs (≥2 absolute fold changes; TPM ≥ 5; FDR *p* ≤ 0.05) for postnatal day (PD) 6.5 compared with PD 4.5 wildtype (green) and *Erβ^KO^* (orange) rat ovaries are shown in the left panels (**A**,**C**,**E**). Differential expression of DEERs, DETFs, and DEDTs for PD 8.5 compared with PD 6.5 wildtype (green) and *Erβ^KO^* rat (orange) ovaries are shown in the right panels (**B**,**D**,**F**). All DEERs, DETFs, and DEDTs were determined based on the TPM values of the transcript variants.

In addition to the quantitative differences in the DEERs and DETFs of the wildtype and *Erβ^KO^* ovaries, we detected distinct differences in the identities of the regulators of gene expression (Figure 9). Only 3 out of 46 DEERs were common between the wildtype and *Erβ^KO^* ovaries on PD 6.5, and 2 out of 8 DEERs were common on PD 8.5 (Figure 9A,B). Similarly, only 3 out of 55 DETFs were common between the wildtype and *Erβ^KO^* ovaries on PD 6.5, and 3 out of 10 DETFs were common on PD 8.5 (Figure 9C,D). The DEDTs also showed a similar pattern; only 99 out of 744 DEDTs were common between the wildtype and *Erβ^KO^* ovaries on PD 6.5, and only 61 out of 191 DEDTs were common on PD 8.5 (Figure 9E,F).

### 2.8. Changes in Transcripts Related to Follicle Assembly

Oocyte nest breakdown results in primordial follicle assembly during early ovarian development [2]. Studies have identified numerous transcription factors and downstream genes involved in this process [2,28,29]. We analyzed the 40 transcript variants of 27 known genes to ascertain if they exhibited a significant differential expression. In PD 6.5 wildtype ovaries, we detected the upregulation of *Irx3-201* and the downregulation of *Nobox-201* and *Akt1-205*. PD 6.5 *Erβ^KO^* ovaries had more interesting patterns as the *Elavl2* and *Notch2* variants displayed conflicting regulations where *Elavl2-201*, *Elavl2-202*, and *Notch2-201* were upregulated and *Notch2-204* and *Elavl2-203* were downregulated. In addition, *Inhba-201* and Akt1-204 were also upregulated and *Irx3-201*, *Irx5-202*, *Nobox-201*, *Figla-201*, and *Sohlh1-201* were also downregulated. We observed the same upregulated and downregulated transcripts in PD 8.5 wildtype ovaries and PD 8.5 *Erβ^KO^* ovaries, showing the same regulation patterns as the PD 6.5 experimental group related to follicle assembly genes.

### 2.9. Changes in Transcripts Related to Primordial Follicle Activation

Primordial follicle activation is a complex process that activates the dormant primordial follicles into primary follicles [30]. Once activated, the follicles progress through successive developmental processes or undergo atresia [31,32]. We analyzed the 58 transcript variants of 34 genes involved in primordial follicle activation. We observed that, compared with PD 4.5 wildtype or *Erβ^KO^* ovaries, the PD 6.5 wildtype or *Erβ^KO^* ovaries showed a similar upregulation of *Igf1* and *Nr5a2* transcript variants. Compared with PD 6.5 wildtype or *Erβ^KO^* ovaries, the PD 8.5 wildtype or *Erβ^KO^* ovaries showed significant downregulation of *Sohlh1*, *Tsc2*, *Hdac*, and *Nobox* transcript variants.

### 2.10. Changes in Transcripts Related to Steroidogenesis

Granulosa cells and theca cells play a crucial role in steroidogenesis [33]. We analyzed the 36 transcript variants of 20 genes involved in steroidogenesis. The transcripts related to steroidogenesis also displayed an altered expression in both wildtype and *Erβ^KO^* ovaries during postnatal development. Although some of the transcript variants of the Hsd gene were upregulated, others were downregulated across the developmental timepoints. Compared with PD 4.5 ovaries, the PD 6.5 wildtype ovaries showed the upregulation of *Hsd17b2-201* and *Hsd3b3-201* and downregulation of *Hsd11b2-201*, *Hsd17b7-201*, *Hsd17b4-203*, and *Hsd3b7-202*. These transcripts also showed a similar expression pattern in PD 6.5 *Erβ^KO^* ovaries compared with PD 4.5 *Erβ^KO^* ovaries. However, we observed that *Hsd17b2-201* was downregulated in PD 8.5 wildtype ovaries. Unlike Hsd, the transcript variants of the *Cyp* and *Star* genes (*Star-201*, *Cyp11a1-201*, *Cyp11a1-202*, *Cyp17a1-201*, and *Cyp19a1-201*) showed a remarkable upregulation in both the wildtype and *Erβ^KO^* ovaries during the postnatal period.

## 3. Discussion

ERβ is a prominent transcriptional regulator in mammalian ovaries. RNA-Seq and ChIP-Seq studies have shown that ERβ regulates the expression of other critical transcriptional regulators in ovaries like NR5A2 [34]. Another study demonstrated that ERβ interacts with ovary-specific transcriptional regulators like FOXL2 to regulate gene expression [35]. Recent studies have also suggested that ERα and ERβ can regulate gene expression through the induction of epigenetic changes [36]. We previously detected that ERβ plays a gatekeeping role in regulating primordial follicle activation [23], and the loss of ERβ accelerates primordial follicle activation in both first-wave follicles (FWFs) as well as second-wave follicles [7]. The increased primordial follicle activation of first-wave primordial follicles in *Erβ^KO^* ovaries becomes evident as early as PD 4.5, while the increased primordial follicle activation of SW primordial follicles becomes apparent as early as PD 8.5 [7,23]. Accordingly, in this study, we collected total RNA from PD 4.5, PD 6.5, and PD 8.5 wildtype ovaries and compared the transcriptomes with those of *Erβ^KO^* ovaries at corresponding timepoints. Our results identified the differential expression of novel transcripts during early ovarian development and elucidated the epigenetic and transcriptional basis of the *Erβ^KO^* phenotype in postnatal ovaries.

**Figure 10:**
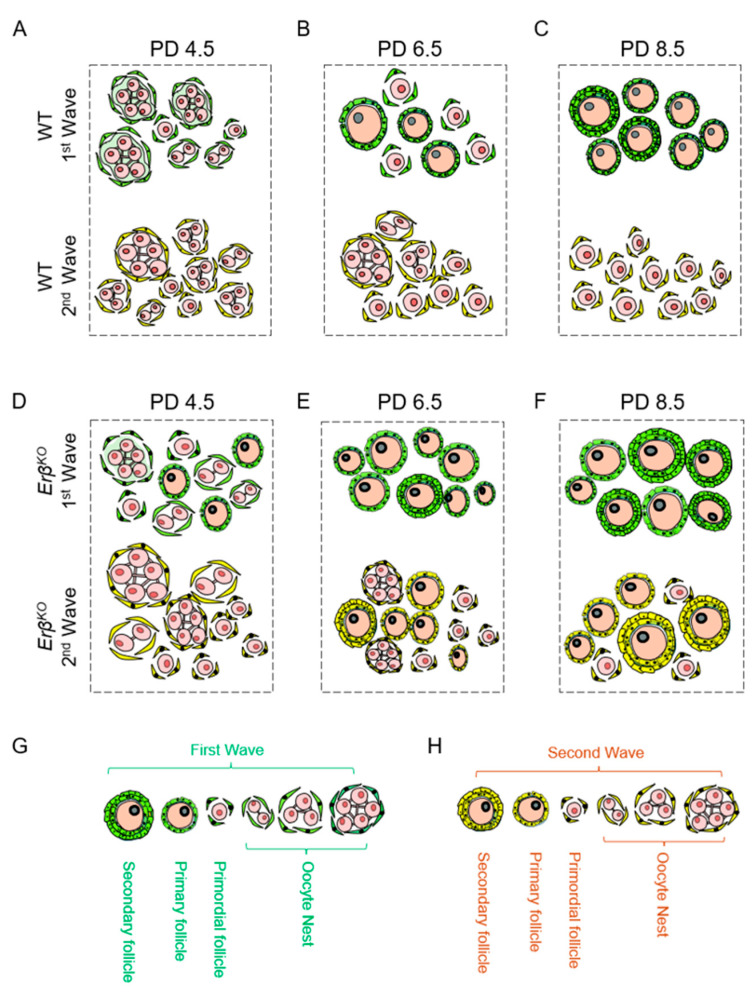
Ovarian follicles of wildtype and *Erβ^KO^* rat ovaries during the postnatal period. Wildtype (WT) PD 4.5 follicles contained activated first-wave follicles, primordial follicles of the first wave, and oocyte nests of both waves (**A**) However, at this timepoint, the second-wave follicles remained as oocyte nests (**A**) The activation of first-wave primordial follicles increased in PD 6.5 and 8.5 wildtype ovaries (**B**,**C**) and was quantitatively greater in *Erβ^KO^* ovaries (**D**–**F**) Although PD 6.5 and 8.5 wildtype ovaries contained primordial follicles of the second wave (**B**,**C**), a portion of the primordial follicles became activated in age-matched *Erβ^KO^* ovaries (**E**,**F**). The lower panel (**G**,**H**) shows a schematic of ovarian follicles in the first wave (**G**) and the second wave (**H**) at different stages of development.

Rodent ovarian follicles develop during the perinatal and early postnatal period [37]. Oocyte nest breakdown and the formation of primordial follicles occur in two successive waves [18,38]. First-wave primordial follicles are formed in the ovarian medulla and are rapidly activated [39]. However, second-wave primordial follicles start assembling in the ovarian cortex at PD 4.5 and are completed by PD 8.5. In contrast to first-wave primordial follicles, second-wave primordial follicles remain dormant until they are recruited for primordial follicle activation [40,41]. The wildtype PD 4.5 follicles contained activated first-wave follicles, primordial follicles of the first wave, and oocyte nests; however, the second-wave follicles remained as oocyte nests (Figure 10A,D). The activation of first-wave primordial follicles increased in the PD 6.5 and 8.5 wildtype ovaries (Figure 10B,C) and was quantitatively greater in *Erβ^KO^* ovaries [7,23] (Figure 10E,F). Although PD 8.5 ovaries contained primordial follicles of the second wave (Figure 10C), a portion of the second wave primordial follicles became activated in the *Erβ^KO^* ovaries [7,23] (Figure 10F). As we performed the transcriptomic analyses on whole ovaries, the results represented the gene expression of both the first- and second-wave follicles.

The transcriptome analyses used in this study represented the transcript variants of the genes expressed in ovarian follicles and interstitial cells. The follicular cells represented many granulosa cells, followed by theca cells and oocytes. We analyzed the changes in epigenetic regulators and transcription factors during early ovarian development in PD 6.5 ovaries compared with PD 4.5 ovaries and in PD 8.5 ovaries compared with PD 6.5 ovaries. We performed similar analyses for wildtype and *Erβ^KO^* ovaries and compared the findings. We further analyzed the DEERs, DETFs, and DEDTs to identify the critical transcripts according to their functional involvement in follicle development and/or steroidogenesis.

We observed that compared with PD 4.5, PD 6.5 wildtype ovaries showed 581 DEDTs, which included 17 DEERs and 23 DETFs. In contrast, age-matched *Erβ^KO^* ovaries expressed 744 DEDTs, including 46 DEERs and 55 DETFs. Subsequently, compared with PD 6.5 ovaries, PD 8.5 wildtype ovaries expressed 920 DEDTs, including 24 DEERs and 68 DETFs. In contrast, age-matched *Erβ^KO^* ovaries expressed only 191 DEDTs, including 8 DEERs and 10 DETFs. It is well known that epigenetic regulators and transcription factors are the upstream regulators of downstream transcripts. Thus, based on these observations, we suggest that when a more significant number of epigenetic or transcriptional regulators are differentially expressed, a more substantial number of DEDTs are produced. Our observations also indicate that a loss of ERβ dysregulates the expression of epigenetic regulators and transcription factors, which alters the expression of downstream genes during neonatal rat ovarian development. Further studies would clarify if ERβ directly or indirectly regulates the DEERs, DETFs, or DEDTs in neonatal rat ovaries.

In addition to the difference in numbers of DEERs, DETFs, and DEDTs during ovarian development between wildtype and *Erβ^KO^* ovaries, the identities of the transcripts were also vastly divergent. Only 7% of DEERs, 5% of DETFs, and 13% of DEDTs were common to PD 6.5 wildtype and *Erβ^KO^* ovaries. Although the proportion of the shared transcripts increased in PD 8.5 ovaries, there were still 25% DEERs, 30% DETFs, and 315 DEDTs between the wildtype and *Erβ^KO^* ovaries. These results suggest that a loss of ERβ in mutant rat ovaries impacts the development of follicular cells from a very early stage. The DEDTs observed between PD 4.5 and PD 6.5 in wildtype ovaries could act on the development of FWFs, while the DEDTs that occur from PD 6.5 to PD 8.5 in the wildtype could act on the development of SWFs. Remarkably, a loss of ERβ resulting in altered DEDTs interfered with the developmental process, leading to the rapid activation of both waves of follicle development in *Erβ^KO^* ovaries.

Although changes in the known epigenetic regulators, transcription factors, and other downstream vital transcripts were observed in the wildtype ovaries (as expected), they were not present in the *Erβ^KO^* ovaries. These findings indicate that a loss of ERβ results in the regulation of gene expression from a very early stage. Although ERβ is predominantly expressed in granulosa cells, a loss of ERβ can be expected to impact the proliferation and differentiation of granulosa cells directly [42,43]. However, bidirectional signaling between granulosa cells and theca cells, and between granulosa cells and oocytes, is well-known in ovarian biology [44,45]. Therefore, it may also be expected that a loss of ERβ in granulosa cells may indirectly impact gene regulation in theca cells and oocytes [10,46].

This study was conducted using whole-body *Erβ^KO^* rat models [47]. However, there are several mouse models, including conditional knockout mice [48,49,50]. Future studies on these mouse models would clarify the role of ERβ in epigenetic and transcription regulation during early ovarian development.

## 4. Materials and Methods

### 4.1. Experimental Model

Wildtype and *Erβ^KO^* Holtzman Sprague-Dawley (HSD; Envigo, Indianapolis, IN, USA) female rats were included in this study. The *Erβ^KO^* rat model was generated by the targeted deletion of exon 3 in the *Erβ* gene, causing a frameshift and null mutation [47]. Rats were screened for the presence of mutations by genotyping PCR using tail-tip or toe-clip DNA samples as previously described [47]. All procedures were performed according to the protocol approved by the University of Kansas Medical Center Animal Care and Use Committee (IACUC).

### 4.2. RNA Sequencing

Ovaries were collected from 4.5, 6.5, and 8.5 day-old wildtype and *Erβ^KO^* female rats. Ovaries were snap-frozen in liquid nitrogen, preserved at −80 °C, and later used for RNA purification [7,23,51]. Total RNA was extracted from the ovaries using TRI Reagent (Millipore-Sigma, St. Louis, MO, USA), and gene expressions were evaluated using RNA sequencing (RNA-Seq) analyses. RNA samples with a RIN value ≥ 9 were used for the library preparation. The total RNA was pooled from 3 independent rats of the same genotype at each postnatal timepoint studied. Then, 500 ng of the pooled total RNA from 3 independent rats was used for each RNA-Seq library preparation using a TruSeq Stranded mRNA kit (Illumina, San Diego, CA, USA) following the manufacturer’s instructions [7]. At each timepoint, 3 RNA-Seq libraries were prepared (representing ovaries from 9 individual rats) and evaluated for quality at the KUMC Genomics Core Facility (Kansas City, KS, USA). RNA sequencing was performed for at least 30 million reads per library using an Illumina HiSeq X sequencer at the Novogene Corporation (Sacramento, CA, USA). The RNA-Seq data were submitted to the Sequencing Read Archive (PRJNA576013 and PRJNA1211271, SRA, NLM). These datasets were analyzed to determine the differential expression of the *Indian Hedgehog* gene in the first wave and the second wave of rat ovarian follicles during ovarian development [7].

### 4.3. Analysis of RNA Sequencing Data

All RNA-Seq data were analyzed using a CLC Genomics Workbench (Qiagen Bioinformatics, Redwood City, CA, USA) as described in our previous publications [11,12]. Clean RNA-Seq reads were obtained by removing low-quality reads and trimming the adapter sequences. The high-quality reads were aligned to the Rattus norvegicus reference genome (mRatBN7.2.111), gene (mRatBN7.2.111_Gene), and mRNA sequences (mRatBN7.2.111_mRNA) using the default parameters [25]. The expression values were measured in TPM, and the values of individual transcript variants (TEs) in whole rat ovaries were determined as described in our previous publications [11,12]. We recently demonstrated the relative advantage of analyzing transcript variants over gene expression (GE) values [25].

### 4.4. Analysis of the Transcript Variants

In this study, we determined the differential expression of mRNA transcript variants as previously described in our publication [25]. The RNA-Seq data were analyzed using the Ensembl *Rattus norvegicus* reference genome (mRatBN7.2.111). The reference sequence had 23,094 genes that expressed 43,831 known transcript variants. This indicated that each gene expressed more than one transcript variant. The transcript variants were indicated by adding a number after the gene name (e.g., the *Mysm1* mRNA transcript variants were *Mysm1-201*, *Mysm1-202*, and *Mysm1-203*).

We analyzed three categories of differentially expressed transcript variants and separated them into three categories (epigenetic regulators, transcription factors, and all the transcript variants indicated as downstream transcripts). A list of 690 epigenetic regulators was prepared according to a recent publication [26]. The 1374 mouse transcription factors list was curated according to human TFs [27]. Each RNA-Seq data file containing TE values was used to generate new tracks containing only the epigenetic regulators or transcription factors, which were used in subsequent analyses.

This study focused on moderately expressed transcripts with TPM values ≥ 5. The threshold *p*-values were selected according to the false discovery rate (FDR) to identify the differentially expressed transcript variants. A transcript variant was considered differentially expressed if the absolute fold change was ≥2 and the FDR *p*-value was ≤0.05 [11,12,52]. The differentially expressed transcript variants were divided into the following three groups: upregulated (≥2 absolute fold changes and an FDR of *p* ≤0.05), downregulated (≤2-fold changes and an FDR of *p* ≤ 0.05), and insignificant (either ≤absolute 2-fold changes and/or an FDR of *p* ≥ 0.05).

Differential expressions of the transcript variants were determined using two different methods. In the first approach, the TE values of PD 6.5 ovaries were compared with those of PD 4.5 ovaries. Then, the TE values of PD 8.5 ovaries were compared with those of PD 6.5 ovaries within the wildtype or the *Erβ^KO^* group. In the second approach, the differentially expressed transcript variants (DEERs, DETFs, and DEDTs) in PD 6.5 or PD 8.5 wildtype ovaries were compared with those of the *Erβ^KO^* ovaries.

### 4.5. Statistical Analysis

Each RNA-Seq library was prepared using pooled RNA samples from three individual wildtype or *Erβ^KO^* rats. Each group of RNA sequencing data consisted of three to four different libraries. For RNA-Seq, each study group contained three library samples. The ’differential expression for RNA-Seq tool’ of the CLC Genomics Workbench calculates multi-factorial statistics using a set of expression tracks based on a negative binomial generalized linear model (GLM). The final GLM fit and dispersion estimate calculates the total likelihood of the model, given the data and the uncertainty of each fitted coefficient. Two statistical tests—the Wald and the likelihood ratio—use one of these values. The across groups (ANOVA-like) comparison uses the likelihood ratio test.

## 5. Conclusions

We analyzed the transcriptome of the whole ovary during the postnatal period to identify the mRNA transcripts involved in early ovarian development. We emphasized the epigenetic regulators and transcription factors to understand the mechanism of downstream transcript expression. Our observations indicate that a loss of ERβ dysregulates the epigenetic regulators and transcription factors in *Erβ^KO^* ovaries, which disrupts the downstream transcripts in ovarian follicles and increases primordial follicle activation.

## Data Availability

SRA; NCBI.

## Supplementary Materials

The following supporting information can be downloaded at: https://www.mdpi.com/article/10.3390/ijms26020760/s1.
