# Supplementary material for: Altered Expression of Epigenetic and Transcriptional Regulators in ERβ Knockout Rat Ovaries During Postnatal Development
Source: Int J Mol Sci. 2025 Jan 17;26(2):760. doi: 10.3390/ijms26020760 (PMC11765817; doi:10.3390/ijms26020760)
Supplement: Supplementary file 1 [file ijms-26-00760-s001.zip › ijms-3344554-supplementary.pdf]

S.1. Epigenetic Regulators

Supplementary Table S1-1. Differentially expressed epigenetic regulators in PD 6.5 wildtype ovaries compared to PD 4.5 wildtype ovaries.

Name	Chrom	Max group mean	Fold change	FDR p-value	ENSEMBL
Morf4l1-201	8	30.77	2,917.98	3.98E-04	ENSRNOT00000080421
Actb-204	12	5.6	614.8	0.01	ENSRNOT00000116486
Phf2-201	17	5.13	47.81	1.35E-07	ENSRNOT00000022669
Msl1-201	10	12.52	3.12	1.69E-03	ENSRNOT00000012812
Mysm1-201	5	13.68	2.37	1.63E-03	ENSRNOT00000039554
Chrac1-201	7	51.61	2.09	4.11E-08	ENSRNOT00000012121
Phf14-208	4	5.77	2.01	1.15E-04	ENSRNOT00000108109
Phf8-202	X	7.33	-2.08	0.02	ENSRNOT00000093422
Glyr1-202	10	26.89	-2.6	2.87E-06	ENSRNOT00000095486
Hdac6-203	X	13.57	-2.72	5.88E-03	ENSRNOT00000116471
Bptf-202	10	5.38	-3.11	4.29E-05	ENSRNOT00000098328
Chd6-202	3	10.67	-5.36	6.56E-06	ENSRNOT00000089958
Ogt-203	X	44.21	-5.92	1.26E-06	ENSRNOT00000100805
Msl1-202	10	6.37	-128.82	5.32E-04	ENSRNOT00000101887
Setd2-201	8	6.07	-148.22	4.90E-07	ENSRNOT00000028409
Kat7-204	10	6.24	-278.56	4.74E-05	ENSRNOT00000109616

Supplementary Table S1-2. Differentially expressed epigenetic regulators in PD 6.5 Erβ^{KO} ovaries compared to PD 4.5 Erβ^{KO} ovaries.

Name	Chrom	Max group mean	Fold change	FDR p-value	ENSEMBL
Pogz-201	2	5.61	342.82	1.79E-07	ENSRNOT00000082849
Kmt2c-203	4	5.63	19.88	2.31E-24	ENSRNOT00000103015
Supt16h-201	15	29.65	11.25	4.67E-06	ENSRNOT00000016288
Hdac3-201	18	11.4	8.21	0.01	ENSRNOT00000060417
Glyr1-204	10	5.43	7.14	5.98E-03	ENSRNOT00000106608
Btaf1-205	1	5.18	4.79	1.59E-05	ENSRNOT00000113933
Smarca1-202	X	6.98	4.18	6.08E-03	ENSRNOT00000114801
Nsd1-205	17	21.12	3.03	8.78E-13	ENSRNOT00000118233
Huwe1-203	X	29.47	3.01	1.92E-14	ENSRNOT00000098599
Dzip3-201	11	16.55	2.95	3.98E-06	ENSRNOT00000002678
Ino80d-201	9	8.86	2.87	0.02	ENSRNOT00000035879
Mysm1-201	5	13.61	2.66	1.31E-04	ENSRNOT00000039554
Jmjd1c-202	20	9.46	2.59	1.31E-05	ENSRNOT00000099911
Hdac3-203	18	36.39	-2.01	1.06E-05	ENSRNOT00000084735
Kdm2a-202	1	37.55	-2.03	2.27E-11	ENSRNOT00000098321
Zmym3-204	X	18.9	-2.04	3.75E-03	ENSRNOT00000076709
Ncor2-205	12	32.93	-2.12	1.96E-06	ENSRNOT00000104620
Prkag2-203	4	9.92	-2.31	0.04	ENSRNOT00000106083
Brd8-207	18	19.83	-2.44	0.05	ENSRNOT00000118939
Ubr5-201	7	25.59	-3.71	1.12E-25	ENSRNOT00000009115
Kdm3b-203	18	15.74	-3.83	8.78E-13	ENSRNOT00000111068
Dpf1-203	1	5.3	-9.51	0.04	ENSRNOT00000108923
Rnf40-203	1	5.6	-12	7.68E-03	ENSRNOT00000098677
Tp53bp1-203	3	9.84	-14.92	3.07E-06	ENSRNOT00000107143
Ogt-203	X	100.89	-97.48	2.80E-29	ENSRNOT00000100805
Tp53bp1-202	3	8.25	-175.1	4.77E-16	ENSRNOT00000106843
Ppp4r3a-204	6	8.3	-290.47	3.68E-10	ENSRNOT00000114004
Setd2-201	8	11.56	-709.46	1.89E-19	ENSRNOT00000028409

Supplementary Table S1-3. Differentially expressed epigenetic regulators in PD 8.5 wildtype ovaries compared to PD 6.5 wildtype ovaries.

Name	Chrom	Max group mean	Fold change	FDR p-value	ENSEMBL
Kat7-204	10	6.46	322.85	1.19E-06	ENSRNOT00000109616
Kdm6a-205	X	8.03	3.02	6.15E-03	ENSRNOT00000109873
Hdac6-203	X	13.11	2.84	7.72E-05	ENSRNOT00000116471
Chd3-204	10	9.2	2.66	5.32E-12	ENSRNOT00000114886
Chd9-202	19	5.79	2.28	1.32E-06	ENSRNOT00000076138
Glyr1-202	10	20.62	2.11	5.74E-05	ENSRNOT00000095486
Sfpq-201	5	129.18	2.01	2.89E-29	ENSRNOT00000084202
Cbx6-201	7	20.8	2.01	4.29E-03	ENSRNOT00000068033
Setdb1-203	2	8.44	-2.04	1.50E-06	ENSRNOT00000111803
Morf4l2-207	X	5.74	-2.06	7.96E-05	ENSRNOT00000116026
Ube2b-202	10	31.35	-2.14	3.06E-04	ENSRNOT00000106084
Ogt-202	X	200.37	-2.23	2.18E-62	ENSRNOT00000082967
Phf14-208	4	5.77	-2.62	7.36E-04	ENSRNOT00000108109
Atxn7-201	15	14.75	-2.8	3.20E-07	ENSRNOT00000010103
Hdgf-202	2	16.18	-326.91	1.19E-06	ENSRNOT00000065211
Actb-204	12	5.6	-649.91	5.00E-03	ENSRNOT00000116486
Actl6a-202	2	17.24	-2,068.55	4.27E-03	ENSRNOT00000098294
Morf4l1-201	8	30.77	-3,077.06	2.57E-03	ENSRNOT00000080421

Supplementary Table S1-4. Differentially expressed epigenetic regulators in PD 6.5 Erβ^{KO} ovaries compared to PD 4.5 Erβ^{KO} ovaries.

Name	Chrom	Max group mean	Fold change	FDR p-value	ENSEMBL
Morf4l1-201	8	35.45	3,009.70	7.40E-03	ENSRNOT00000080421
Mta1-201	6	12.48	2.36	0.04	ENSRNOT00000006521
Ubr5-201	7	14.12	2.07	4.97E-04	ENSRNOT00000009115
Chd6-202	3	10	-2.29	0.03	ENSRNOT00000089958
Hdac3-201	18	11.4	-5.36	0.02	ENSRNOT00000060417
Jade1-202	2	5.49	-41.27	1.69E-06	ENSRNOT00000098865
Taf9b-201	X	5.9	-1,053.93	0.01	ENSRNOT00000090833

S.2. Transcription Factors

Supplementary Table S2-1. Top 5 upregulated and downregulated, differentially expressed transcription factors in PD 6.5 wildtype ovaries compared to PD 4.5 wildtype ovaries.

Name	Chrom	Max group mean	Fold change	FDR p-value	ENSEMBL
Zfp518a-204	1	8.09	16.39	2.12E-07	ENSRNOT00000105629
Foxk2-203	10	8.64	5.27	0.05	ENSRNOT00000099287
Zfp711-202	X	5.48	4.31	6.11E-03	ENSRNOT00000095292
Chchd3-204	4	24.36	4.25	8.09E-05	ENSRNOT00000107634
Zbed4-201	7	6.87	2.99	6.25E-03	ENSRNOT00000006050
Rbck1-203	3	17.55	-3.8	5.46E-04	ENSRNOT00000085995
Prr12-202	1	7.14	-29.13	7.11E-11	ENSRNOT00000094541
Tgif1-202	9	5.79	-77.26	2.32E-03	ENSRNOT00000102373
Kat7-204	10	6.24	-277.85	1.49E-05	ENSRNOT00000109616
Mlx-204	10	5.05	-491.94	0.02	ENSRNOT00000119770

Supplementary Table S2-2. Top 5 upregulated and downregulated, differentially expressed transcription factors in PD 6.5 Erβ^{KO} ovaries compared to PD 4.5 Erβ^{KO} ovaries.

Name	Chrom	Max group mean	Fold change	FDR p-value	ENSEMBL
Zfp786-201	4	5.5	869.67	0.01	ENSRNOT00000038550
Mlx-202	10	6.56	698.55	0.02	ENSRNOT00000099833
Nfx1-201	5	14.33	9.34	2.04E-12	ENSRNOT00000060691
Glyr1-204	10	5.43	7.16	0.01	ENSRNOT00000106608
Son-201	11	34.05	6.27	4.45E-06	ENSRNOT00000002769
Nr1d2-203	15	18.92	-4.77	0.02	ENSRNOT00000106171
Dpf1-203	1	5.3	-9.38	0.03	ENSRNOT00000108923
Zbtb7c-202	18	6	-12.33	0.02	ENSRNOT00000101836
Zfp260-202	1	6.97	-141.72	1.46E-09	ENSRNOT00000074301
Zfp384-203	4	5.92	-1,163.82	6.71E-03	ENSRNOT00000036654

Supplementary Table S2-3. Top 5 upregulated and downregulated, differentially expressed transcription factors in PD 8.5 wildtype ovaries compared to PD 6.5 wildtype ovaries.

Name	Chrom	Max group mean	Fold change	FDR p-value	ENSEMBL
Mlx-204	10	9.31	1,075.75	2.97E-03	ENSRNOT00000119770
Kat7-204	10	6.46	327.56	2.37E-08	ENSRNOT00000109616
Fosl2-201	6	14.08	5.84	4.08E-06	ENSRNOT00000107475
Pparg-202	4	11.18	3.67	2.42E-07	ENSRNOT00000082969
Foxo1-201	2	77.47	3.5	1.02E-36	ENSRNOT00000018244
Lhx9-201	13	28.68	-5.35	3.09E-33	ENSRNOT00000014218
Lhx9-203	13	6.82	-5.46	3.26E-05	ENSRNOT00000116473
Ets1-204	8	12.83	-6.19	1.27E-08	ENSRNOT00000102138
Nfe2l2-203	3	21.52	-20.96	3.12E-12	ENSRNOT00000110389
Crem-210	17	6.1	-1,627.15	5.15E-03	ENSRNOT00000115950

Supplementary Table S2-4. Top 5 upregulated and downregulated, differentially expressed transcription factors in PD 8.5 Erβ^{KO} ovaries compared to PD 6.5 Erβ^{KO} ovaries.

Name	Chrom	Max group mean	Fold change	FDR p-value	ENSEMBL
Zbtb7c-202	18	9.55	37.5	3.66E-09	ENSRNOT00000101836
Zfp410-204	6	7.23	9.89	0.04	ENSRNOT00000109998
Eea1-202	7	5.43	9.65	7.95E-03	ENSRNOT00000087297
Camta2-203	10	5.24	6.75	0.04	ENSRNOT00000109888
L3mbtl4-201	9	6.37	2.08	0.05	ENSRNOT00000029210
Eea1-206	7	11.8	-2.15	0.04	ENSRNOT00000117665
Hoxc6-202	7	5.93	-2.52	0.04	ENSRNOT00000107142
Tead3-203	20	9.78	-8.09	5.47E-03	ENSRNOT00000107495
Zbed3-201	2	10.19	-16.55	2.91E-03	ENSRNOT00000040983
Nfe2l2-203	3	11.16	-18.09	2.60E-06	ENSRNOT00000110389

S.3. Downstream Genes

Supplementary Table S3-1. Top 10 upregulated and downregulated, differentially expressed downstream transcripts in PD 6.5 wildtype ovaries compared to PD 4.5 wildtype ovaries.

Name	Chrom	Max group mean	Fold change	FDR p-value	ENSEMBL
Macf1-202	5	6.28	10,571.87	6.89E-04	ENSRNOT00000081482
Actr3-201	13	29.64	4,907.80	3.76E-03	ENSRNOT00000004520
Tcof1-203	18	8.76	3,271.29	3.53E-04	ENSRNOT00000115228
Morf4l1-201	8	30.77	3,025.94	3.96E-04	ENSRNOT00000080421
Pmp22-201	10	21.24	2,413.27	6.83E-03	ENSRNOT00000041606
Mdh2-202	12	28.28	2,361.01	6.82E-04	ENSRNOT00000112030
Faf1-203	5	14.82	2,070.14	8.30E-03	ENSRNOT00000108187
Serpinb9-204	17	11.89	1,804.43	0.01	ENSRNOT00000115382
Nnt-205	2	5.18	1,145.68	3.28E-03	ENSRNOT00000112693
Pim3-202	7	5.73	999.55	0.03	ENSRNOT00000085835
Ppie-203	5	5.61	-369.39	0.03	ENSRNOT00000094173
Mlx-204	10	5.05	-486.77	0.01	ENSRNOT00000119770
Clip1-206	12	6.01	-504.29	1.10E-06	ENSRNOT00000098458
Uckl1-207	3	5.35	-507.19	0.02	ENSRNOT00000113965
Bet1-202	4	8.54	-586.67	1.78E-03	ENSRNOT00000106013
Smc3-201	1	5.35	-1,143.52	1.77E-04	ENSRNOT00000019560
Lrrc8d-205	14	6.45	-1,313.96	2.62E-03	ENSRNOT00000109682
Slc25a36-202	8	19.04	-1,863.43	5.83E-04	ENSRNOT00000110158
Znf740-201	7	10.29	-2,061.19	1.95E-03	ENSRNOT00000016240
Spint2-201	1	38.16	-2,330.83	3.61E-04	ENSRNOT00000028006

Supplementary Table S3-2. Top 10 upregulated and downregulated, differentially expressed downstream transcripts in PD 8.5 wildtype ovaries compared to PD 6.5 wildtype ovaries.

Name	Chrom	Max group mean	Fold change	FDR p-value	ENSEMBL
Rbmx-201	X	14.39	1,910.64	2.02E-03	ENSRNOT00000001154
Znf740-201	7	6.78	1,662.01	2.94E-03	ENSRNOT00000016240
Smc3-201	1	6.46	1,646.93	1.26E-04	ENSRNOT00000019560
Slc4a2-202	4	5.76	1,502.93	5.89E-05	ENSRNOT00000108170
Arpc5-204	13	12.24	1,372.79	8.10E-05	ENSRNOT00000107757
Mlx-204	10	9.31	1,091.39	2.18E-03	ENSRNOT00000119770
Spint2-201	1	12.36	919.26	2.98E-03	ENSRNOT00000028006
Zfp689-206	1	5.01	822.61	3.83E-04	ENSRNOT00000024969
Clip1-206	12	5.53	524.86	1.73E-07	ENSRNOT00000098458
Alad-202	5	6.38	515.47	1.69E-03	ENSRNOT00000075916
Nnt-205	2	5.18	-1,152.25	9.58E-03	ENSRNOT00000112693
Eif3e-201	7	12.55	-1,360.94	6.65E-04	ENSRNOT00000038340
Nusap1-203	3	9.22	-1,404.17	6.17E-03	ENSRNOT00000095179
Txnl1-204	18	19.99	-1,405.49	7.00E-04	ENSRNOT00000100860
Crem-210	17	6.1	-1,609.51	5.85E-03	ENSRNOT00000115950
Rbm25-201	6	8.87	-1,686.68	6.99E-03	ENSRNOT00000003834
Serpinb9-204	17	11.89	-1,833.40	4.26E-03	ENSRNOT00000115382
Sypl1-203	6	15.38	-2,017.26	5.16E-03	ENSRNOT00000097655
Actl6a-202	2	17.24	-2,046.37	3.72E-03	ENSRNOT00000098294
Morf4l1-201	8	30.77	-3,035.09	2.79E-03	ENSRNOT00000080421

Supplementary Table S3-3. Top 10 upregulated and downregulated, differentially expressed downstream transcripts in PD 6.5 Erβ^{KO} ovaries compared to PD 4.5 Erβ^{KO} ovaries.

Name	Chrom	Max group mean	Fold change	FDR p-value	ENSEMBL
Srrm2-201	10	38.7	18,234.56	1.65E-07	ENSRNOT00000091287
Macf1-202	5	5.04	7,047.30	4.14E-06	ENSRNOT00000081482
Cdc42bpa-206	13	23.58	3,425.58	1.18E-18	ENSRNOT00000116713
Clasp2-205	8	5.67	2,104.65	2.90E-03	ENSRNOT00000107377
Ephx1-203	13	17.87	1,826.48	4.13E-03	ENSRNOT00000085279
Strbp-202	3	13.53	1,660.51	5.38E-03	ENSRNOT00000087611
Fam168a-203	1	6.27	1,625.53	5.90E-03	ENSRNOT00000097710
Lrrc8d-205	14	7.03	1,494.84	5.28E-03	ENSRNOT00000109682
Mex3d-201	7	8.83	1,263.67	8.32E-03	ENSRNOT00000040170
Sik2-202	8	7.94	1,059.07	3.61E-13	ENSRNOT00000091732
Eng-204	3	9.79	-2,323.79	1.24E-04	ENSRNOT00000114060
Slc22a17-202	15	22.79	-2,381.63	2.63E-03	ENSRNOT00000102200
Ube3b-204	12	8.63	-2,514.75	2.33E-03	ENSRNOT00000116293
Carmil1-201	17	10.66	-3,016.88	1.58E-03	ENSRNOT00000059510
Larp1b-202	2	18.94	-3,469.11	1.17E-03	ENSRNOT00000090861
Nectin2-203	1	25.14	-3,508.81	1.24E-03	ENSRNOT00000117594
Ube4b-202	5	15.09	-4,021.84	9.37E-04	ENSRNOT00000105982
Stard9-201	3	5.49	-4,877.36	5.82E-04	ENSRNOT00000048141
Prkacb-202	2	22.51	-5,311.88	4.87E-04	ENSRNOT00000109432
Col1a1-203	10	163.55	-53,077.70	2.87E-06	ENSRNOT00000115096

Supplementary Table S3-4. Top 10 upregulated and downregulated, differentially expressed downstream transcripts in PD 8.5 Erβ^{KO} ovaries compared to PD 6.5 Erβ^{KO} ovaries.

Name	Chrom	Max group mean	Fold change	FDR p-value	ENSEMBL
Morf4l1-201	8	35.45	2,995.97	6.42E-03	ENSRNOT00000080421
Prkacb-202	2	10.02	2,519.69	8.45E-03	ENSRNOT00000109432
Nectin2-203	1	10.45	1,558.51	0.02	ENSRNOT00000117594
Arpc5-204	13	14.96	1,542.52	0.03	ENSRNOT00000107757
Exoc2-205	17	5.76	1,522.67	0.02	ENSRNOT00000092101
Slc35a1-201	5	10.19	1,167.15	6.03E-03	ENSRNOT00000011969
Ifrd2-203	8	9.71	1,044.36	0.03	ENSRNOT00000112128
Ociad1-202	14	15.62	926.17	0.03	ENSRNOT00000094276
Slc22a17-202	15	8.19	914.7	0.04	ENSRNOT00000102200
Rab4a-202	19	6.09	498.21	0.02	ENSRNOT00000098153
RGD1565355-201	4	5.63	-455.94	0.02	ENSRNOT00000008319
Arih1-202	8	5.88	-467.69	0.02	ENSRNOT00000084758
Rdh11-203	6	6.54	-565.73	0.01	ENSRNOT00000105155
Brms1-202	1	8.24	-661.13	0.04	ENSRNOT00000072647
Fap-203	3	5.42	-765.52	0.04	ENSRNOT000096836
Rab35-203	12	6.74	-798.79	0.03	ENSRNOT00000106644
Exosc9-203	2	8.84	-808.62	0.03	ENSRNOT00000108461
Taf9b-201	X	5.9	-1,069.45	6.43E-03	ENSRNOT00000090833
P2rx1-203	10	8.31	-1,168.29	3.37E-03	ENSRNOT00000118157
Akt2-202	1	15.53	-1,503.78	0.02	ENSRNOT00000100544
